# Crossmatch: Alloimmunization versus unspecific agglutination reactions?

**DOI:** 10.1111/jvim.16626

**Published:** 2023-01-04

**Authors:** Urs Giger

**Affiliations:** ^1^ DACVIM (Small Animal Internal Medicine), DECVIM‐CA (Internal Medicine), DECVCP (Clinical Pathology) Zürich Switzerland

Dear Editors,

Dr Barbara Kohn's group at the Clinic for Small Animals of the Freie University Berlin, Germany, recently reported on crossmatch results in (pre‐) transfused dogs in *JVIM*.^1^ These authors used a tube crossmatch method without canine antiglobulins to assess the potential appearance of alloantibodies posttransfusion. They reported that a subset of 21 transfused tested dogs was exhibiting “very weak” microscopic agglutination reactions before and within hours to days after transfusion. These responses were nonrising over time and often disappeared rapidly after blood transfusion. Based on these data, they claim that alloantibodies developed within hours to days posttransfusion and recommend that all dogs requiring transfusion should be crossmatched at day 1, instead of the current practice of crossmatching 4 days posttransfusion.^1^

Based upon longstanding principles in basic immunology and transfusion medicine and my own experience with blood typing, crossmatching, and alloimmunization in companion animals over the past decades, I would like to comment on the authors' claims and recommendations for the following reasons:

- Neither in vitro nor in vivo immunological data were presented by Kohn et al^1^ to recommend routine crossmatching before or on day 1 after the first transfusion in dogs requiring a second transfusion. In addition, no evidence was provided that the “very weak” agglutination reactions were alloantibody‐mediated.
- Contrary to the authors' hypothesis, there is no scientific evidence that any induced (allo‐) antibodies can ever develop within hours to 4 days in either humans or domestic animals. Indeed, mounting a clinically detectable antibody response to any foreign antigen, including infectious agents and blood groups, requires antigen recognition and activation of specific lymphocytes and subsequent differentiation into effector and memory cells, all of which take time. As such, induced (allo‐) antibodies in any species are not detectable for at least 4 days, and generally require 10 or more days to be detectable.^2^
- Observing agglutination in a crossmatch test without applying any species‐specific antiglobulin reagents cannot be equated with finding alloantibodies. In their tube crossmatch test, the authors simply mixed plasma with RBCs from recipient and donor and certainly did not determine scientifically if this agglutination was antibody‐specific. In human transfusion medicine, the crossmatch tests currently used include antiglobulins (Coombs' reagent) to confirm that agglutination is antibody mediated. Over the past decade, in‐clinic canine antiglobulin‐enhanced crossmatch laboratory tests and kits for dogs have become available and could have been applied to specifically detect alloantibodies in these samples.^3^, ^4^
- Alloimmunization in humans and animals often elicits strong IgM and then IgG agglutinins (and hemolysins) against various blood groups one or more weeks post transfusion which increase in strength and are commonly long‐lasting for months to a lifetime. As alloantibodies are expected to bind to transfused erythrocytes and lead to agglutination before free unbound alloantibodies are found in recipient plasma, it makes it even less likely that unbound alloantibodies could be detected in a crossmatch test so early posttransfusion.
- Interpretation of the authors' tube crossmatch test is difficult, particularly when they only found “very weak” and only microscopic agglutination.^1^ Crossmatch gel column techniques for laboratory use and in‐clinic immunochromatographic strip and gel minitube kits are available in animal blood banking and veterinary transfusion medicine and easier to perform and grade. The authors' observed microscopic agglutination reactions of 1+ to 2+ could easily be unspecific because tube agglutination tests can be affected by many factors including disease state, inferior quality of transfused red blood cells, immediate inflammatory responses in recipients,^5^ condition of donor blood aliquoted in a tube not approved for long‐term storage of blood, and testing methods.,^5^, ^6^, ^7^
- When finding incompatibilities with antiglobulin‐enhanced crossmatch tests in humans, blood group antibody screening with a panel of erythrocytes with different blood types is used to assess alloantibody specificity. While *DEA 1* compatible blood was apparently transfused, the authors did not confirm typing results nor perform extended typing to potentially associate any claimed crossmatch agglutination with blood type incompatibility. Aside from *DEA 1* kits, typing kits for *DEA 4*, *DEA 5*, *Dal*, and *Kai 1/2* as well as typing reagents are commercially available and could have been used in their study.^5^
- These authors also observed “very weak” microscopic agglutination reactions before transfusions, which is unusual and unlikely to be alloantibody‐mediated or clinically relevant. Although there have been few reports of the presence of weak naturally occurring alloantibodies in dogs, for example, *anti‐DEA 7*, an apparently unusual acquired blood type, the data is marginal and is not supported by modern veterinary clinical transfusion practice and clinical experience with transfused dogs over the past decades. No alloantibody‐mediated acute hemolytic transfusion reactions have been documented clinically in dogs that were not previously transfused. However, acute nonimmune hemolysis may occur because of inappropriate collection, processing, storage, and administration of blood.^8^
- These authors also provide no evidence of any delayed hemolytic transfusion reactions because of the development of alloantibodies in the transfused dogs for which in vitro agglutination reactions were detected. While I have very rarely seen a delayed (triggered by the development of new alloantibodies) hemolytic transfusion reactions in dogs (eg, against *Dal*‐antigen*), published clinical evidence of delayed alloantibody‐mediated hemolytic transfusion reactions in dogs is lacking.

In conclusion, I believe the authors' data does not provide specific evidence of alloantibody development before and shortly after transfusion nor does it support the need for recommending crossmatching before 4 days after a first transfusion in dogs. Rather than ignoring or refuting current immunology standard principles, Kohn's study likely exemplifies the unspecificity of a nonantiglobulin enhanced crossmatch and the questionable value of weakly positive crossmatch results. As such, their recommendations may unnecessarily delay and even prevent timely transfusions.

Finally, the continued use of xenotransfusions in cats, which have been known for more than a century to elicit immediately strong incompatibility reactions in vivo, because of the presence of preexisting naturally occurring antierythrocytic xenoantibodies.^9^ should pause anyone to consider “very weak” microscopic agglutination as evidence of any acute alloimmune hemolytic transfusion reaction. Such reactions are completely different from alloantibody responses induced by transfusion of canine blood with an incompatible blood type and clinicians should not automatically conclude that unspecific minor agglutination reactions are clinically relevant, as this would deter transfusion of animals in need of life‐saving transfusions.

## CONFLICT OF INTEREST DECLARATION

The author has been a scientific advisor to various companies involved in blood compatibility testing including Alvedia, DMS Laboratories and some lectures on transfusion medicine at conferences have been supported by companies.

## ENDNOTE

* Watson J, Euler CC, Tappin S, Hale AS, Giger U. Dal‐blood type in breeds beyond Dalmatians: A challenge in a previously transfused Lhasa Apso. Abstract BSAVA Congress proceedings 2022.
